# Tapering and withdrawal of tocilizumab in patients with systemic juvenile idiopathic arthritis in inactive disease: results from an alternative dosing regimen in the TENDER study

**DOI:** 10.1186/1546-0096-12-S1-O13

**Published:** 2014-09-17

**Authors:** F De Benedetti, N Ruperto, H Brunner, A Grom, N Wulffraat, M Henrickson, R Jerath, Y Kimura, AK Kadva, J Wang, A Martini, D Lovell

**Affiliations:** 1 IRCCS Ospedale Pediatrico Bambino Gesú, Rome, Italy; 2 PRINTO, Genoa, Italy; 3 PRCSG, Cincinnati, USA; 4 Genentech, South San Francisco, USA; 5 Roche Products Ltd., Welwyn Garden City, UK

## Introduction

The TENDER clinical trial is a 3-part, 5-year, phase 3 study of tocilizumab (TCZ) in patients with active systemic juvenile idiopathic arthritis (sJIA). After 2 years of treatment, sJIA patients who have maintained clinically inactive disease (CID) for 3 months are given the option to participate in an alternative TCZ dosing regimen aimed at spacing the infusions and eventually withdrawing TCZ.

## Objectives

To describe the patients registered to participate in the optional alternative dosing schedule in the TENDER study.

## Methods

To qualify for the optional alternative dosing schedule, patients had to be in the study for a minimum of 2 years and had to achieve American College of Rheumatology JIA CID status. Among the 112 patients enrolled, 39 (35%) entered the optional alternative dosing regimen. This entailed a staged prolongation of the time interval between TCZ infusions from 2 weeks (standard interval) to 3 weeks, then 4 weeks, with the option of terminating TCZ after the discontinuation of any treatment, including oral corticosteroids, NSAIDs, and MTX (if being treated with MTX).

## Results

Twenty-three male and 16 female patients entered the optional alternative dosing schedule. The mean characteristics of these patients at the start of TCZ treatment were 14.2 active joints, 15.4 joints with limitation of motion, physician global VAS score of 58.5, CHAQ-DI score of 1.62, and erythrocyte sedimentation rate of 56.8. Fifteen had fever. Of these 39 patients, 20 patients lost CID status at different points along the alternative dosing schedule until the data review of May 2014. In these 20 patients, the time to loss of CID status ranged from 1.4 to 27.9 months from initiation of the optional alternative dosing schedule (n = 4 on 3-week dosing; n = 10 on 4-week dosing; n = 6 off TCZ). Risk for losing inactive disease status on the optional alternative dosing schedule was 62.5% (10/16) in patients on concomitant methotrexate and 43.5% (10/23) in patients not on it. During the April 2013 data review, inactive disease status was maintained in 26 of 39 patients (67%), whereas during the May 2014 data review inactive disease status was maintained in 19 of the 39 patients (49%) entering the optional alternative dosing schedule. Dosing intervals were every 3 weeks in 3 patients and every 4 weeks in 9 patients; 7 patients were able to discontinue TCZ (range of time since discontinuation: 13.7-20.8 months). During the April 2013 data review, 9 patients were able to discontinue TCZ. During the May 2014 data review, 7 patients maintained CID status and remained off TCZ, and 2 patients returned to the 2-week dosing interval.

## Conclusion

A proportion of patients with sJIA who maintain clinically inactive disease status can progressively space TCZ infusions. Of the 35% who entered the optional alternative dosing regimen, approximately half were able to maintain inactive disease over an extended period of time.

## Trial registration identifying number

TENDER, NCT00642460

## Disclosure of interest

F. De Benedetti Grant / Research Support from: Abbott, Pfizer, BMS, Roche, Novimmune, Novartis, S0BI, N. Ruperto Grant / Research Support from: Abbott, AstraZeneca, BMS, Centocor, Eli Lilly, Francesco Angelini s.p.a., GlaxoSmithKline, Italfarmaco, Merck Serono, Novartis, Pfizer, Regeneron, Roche, Sanofi Aventis, Schwarz Biosciences, Xoma, Wyeth, Consultant for: Abbott, AstraZeneca, BMS, Centocor, Eli Lilly, Francesco Angelini s.p.a., GlaxoSmithKline, Italfarmaco, Merck Serono, Novartis, Pfizer, Regeneron, Roche, Sanofi Aventis, Schwarz Biosciences, Xoma, Wyeth, H. Brunner Consultant for: Novartis, Genentech, MedImmune, EMD Serono, AMS, Pfizer, UCB, Janssen, Speaker Bureau of: Genentech, A. Grom Grant / Research Support from: Roche, Consultant for: Novartis, N. Wulffraat Grant / Research Support from: Novartis, Roche, Pfizer, AbbVie, Consultant for: Novartis, Roche, Pfizer, M. Henrickson: None declared., R. Jerath: None declared., Y. Kimura: None declared., A. Kadva Employee of: Genentech, a member of the Roche group, J. Wang: None declared., A. Martini Grant / Research Support from: Abbott, AstraZeneca, BMS, Centocor, Eli Lilly, Francesco Angelini s.p.a., GlaxoSmithKline, Italfarmaco, Merck Serono, Novartis, Pfizer, Regeneron, Roche, Sanofi Aventis, Schwarz Biosciences, Xoma, Wyeth, Consultant for: Abbott, AstraZeneca, BMS, Centocor, Eli Lilly, Francesco Angelini s.p.a., GlaxoSmithKline, Italfarmaco, Merck Serono, Novartis, Pfizer, Regeneron, Roche, Sanofi Aventis, Schwarz Biosciences, Xoma, Wyeth, D. Lovell Consultant for: AstraZeneca, Centocor, Janssen, Wyeth, Amgen, BMS, Abbott, Pfizer, Regeneron, Hoffmann La-Roche, Novartis, Genentech, Speaker Bureau of: Genentech, Roche.

